# Meta-analysis of effects of yoga exercise intervention on sleep quality in breast cancer patients

**DOI:** 10.3389/fonc.2023.1146433

**Published:** 2023-06-30

**Authors:** Jingzhi Zhu, Xiaojuan Chen, Xinxian Zhen, Huan Zheng, Hong Chen, Huimin Chen, Huizhao Liao, Jinru Zhu, Chaoyu Wang, Zhenzhen Zheng, Riken Chen, Yang Wang

**Affiliations:** ^1^South China Research Center for Acupuncture and Moxibustion, Medical College of Acupuncture Moxibustion and Rehabilitation, Guangzhou University of Chinese Medicine, Guangzhou, Guangdong, China; ^2^Medical College, Jiaying University, Meizhou, Guangdong, China; ^3^Department of Respiratory and Critical Care Medicine, Taishan People's Hospital, Jiangmen, Guangdong, China; ^4^Department of Endocrinology, The Second Affiliated Hospital of Guangdong Medical University, Zhanjiang, Guangdong, China; ^5^Department of Respiratory and Critical Care Medicine, The People’s Hospital of Yubei, District of Chongqing City, Chongqing, China; ^6^Department of Traditional Chinese Medicine, The Second Affiliated Hospital of Guangdong Medical University, Zhanjiang, Guangdong, China; ^7^Guangzhou Medical University, State Key Laboratory of Respiratory Disease, National Clinical Research Center for Respiratory Disease, Guangzhou Institute of Respiratory Health, The First Affiliated Hospital of Guangzhou Medical University, Guangzhou, Guangdong, China; ^8^Department of Respiratory and Critical Care Medicine, The Second Affiliated Hospital of Guangdong Medical University, Zhanjiang, Guangdong, China; ^9^Department of Respiratory and Critical Care Medicine, Taishan Hospital of Traditional Chinese Medicine, Jiangmen, Guangdong, China; ^10^Department of Respiratory and Critical Care Medicine, The People’s Hospital of Chongqing Liang Jiang New Area, Chongqing, China

**Keywords:** yoga, exercise, breast cancer, sleep quality, meta-analysis

## Abstract

**Objective:**

This study seeks to systematically evaluate and test the effects of yoga exercise intervention programs on sleep quality in breast cancer patients in order to suggest more optimized exercise programs.

**Method:**

Computer searches of the PubMed, Embase, Cochrane Library, Web of Science and CINAHL databases are conducted from the date of their inception to June 8^th^, 2022 to collect randomized controlled trials on the effects of yoga exercise intervention on sleep quality in breast cancer patients. Two investigators independently carry out the inclusion and exclusion criteria literature screening, data extraction and methodological quality assessment of the included literature by applying the Cochrane risk of bias tool. Subgroup analysis is performed using RevMan 5.4.1 software, and the six moderating variables of intervention format, intervention type, weekly intervention frequency, total intervention duration, single intervention duration and intervention evaluation at different time points are set for the 782 subjects of the 12 included publications.

**Results:**

Twelve randomized controlled trials with a total sample size of 782 subjects are included, including 393 subjects in the experimental group and 389 subjects in the control group. The meta-analysis shows that yoga exercise intervention is effective in improving sleep quality in breast cancer patients [SMD = -0.40, 95% CI: (-0.71, -0.09), P = 0.01]; yoga exercise intervention focusing on positive meditation [SMD = -0.55, 95% CI: (-1.08, -0.03), P = 0.04] is effective in improving sleep; yoga exercise intervention two or three times a week is effective in improving sleep quality [SMD = -0.69, 95% CI: (-1.19, -0.19), P = 0.007]; yoga exercise intervention for 6–8 weeks significantly improves sleep quality [SMD = -0.86, 95% CI: (-1.65, -0.08), P =0.03]; and evaluation immediately after the end of intervention improves sleep outcomes [SMD = -0.17, 95% CI: (-0.33, 0.00), P = 0.05], while differences in sleep quality improvement are not statistically significant for the remaining subgroup outcomes (P > 0.05).

**Conclusion:**

The available evidence suggests that yoga exercise intervention has good effects on improving sleep quality in breast cancer patients. Positive meditation intervention type, intervention frequency of two or three times per week, total intervention duration of 6–8 weeks and evaluation immediately after the end of intervention are shown to be effective in improving sleep quality.

## 1 Introduction

Breast cancer is the most common malignancy and the leading cause of cancer death in women worldwide (1), with an estimated 2.3 million new cases and 685,000 deaths in 2020. New breast cancer cases are expected to reach 4.4 million in 2070. The incidence of breast cancer is increasing by 5% per year in low and middle-income countries, and is therefore an increasingly urgent public health problem (2). Surgery combined with adjuvant chemotherapy is currently the most common clinical treatment as it significantly improves the prognosis of breast cancer patients (3), but it also brings a series of adverse effects, most frequently sleep disorders. Matthews et al. showed a high incidence of sleep disorders in breast cancer patients of up to 80% (4). Sleep disorders can impair patients’ immune systems, cognitive abilities and daily functions, and affect their mood, as well as being closely related to tumor development and progression (5). However, there is no standard effective treatment for the improvement of sleep quality, and medication remains the most common treatment (6), but hypnotics are associated with many side effects such as drowsiness, cognitive impairment, dependence, tolerance and poor long-term efficacy. This includes long-term use of benzodiazepines, which correspond to a greater risk of fractures, cognitive decline, and dependence. Non benzodiazepine receptor agonists (zolpidem) are associated with rare but severe adverse reactions, including dementia, delirium, sleepwalking, severe injury, increased risk of fractures, and cancer (7).

A study by Collins et al. showed that yoga has been identified to play a role in improving sleep (8). It was recently suggested that yoga practice can improve overall sleep efficiency and total sleep time (9). The beneficial effects of yoga exercise on sleep are being increasingly recognized. Accordingly, yoga exercise has the same potential to improve sleep quality in breast cancer patients, and studies on the effects of yoga on sleep disorders in breast cancer patients have been conducted in recent years, including Carson and Jong et al. Danhauer et al. found that yoga can improve the quality of sleep and reduce the potential of breast cancer treatment related symptoms, and there is no side effect of sleeping pills to alleviate the pain of patients. However, different intervention variables (intervention format, intervention type, weekly intervention frequency, total intervention duration, single intervention duration and intervention evaluation at different time points) were used for yoga exercise intervention in these studies, resulting in large differences in intervention effects across studies and a lack of consistency in clinical protocols, which has caused great concern among clinical practitioners.

Therefore, it is necessary to conduct a meta-analysis of the effects of yoga exercise intervention across different intervention variables on the sleep quality of breast cancer patients in order to provide the best evidence-based foundation for the effective improvement of patients’ sleep.

## 2 Material and methods

### 2.1 Search strategy

Computer searches of the PubMed, Embase, Cochrane Library, Web of Science and CINAHL databases were conducted from their inception to June 8^th^, 2022. Detections include PubMed(Date of inception 1940s - June 8, 2022), Embase(Date of inception 1960 – June 8, 2022), Cochrane Library(Date of inception 1999 – June 8, 2022), Web of Science(Date of inception 1975 – June 8, 2022), CINAHL(Date of inception 1982 – June 8, 2022) etc. Topics were split into yoga exercise, breast cancer patients, sleep quality and randomized controlled trials (RCTs) according to the PICOS principles: (1) P (participant), English search terms for breast cancer patients: Breast Neoplasm, Breast Tumor, Breast Cancer, Mammary Cancer, Malignant Neoplasm of Breast, Human Mammary Neoplasm, Breast Carcinoma; (2) I (intervention), English search terms for yoga exercise: yoga, yogic, asana, pranayama, yoga exercise; (3) O (outcomes), English search terms for sleep quality: sleep quality, sleep; and (4) S (study design), English search terms for randomized controlled trials: randomized controlled trial, controlled clinical trial, randomized, placebo, randomly. The search was conducted using a combination of MeSH subject terms and free terms, and was adjusted to the characteristics of each database. Each search term was linked with “OR”, and the four sets of results were linked with the Boolean operator “AND” to search for relevant literature.

### 2.2 Inclusion and exclusion criteria

#### 2.2.1 Inclusion criteria

Criteria were developed according to the PICOS principles. The inclusion criteria were as follows: (1) subjects: patients aged ≥ 18 years with a pathological diagnosis of breast cancer; (2) intervention: yoga exercise intervention in the experimental group and unlimited care measures in the control group; (3) study type: RCT; (4) outcome indicators: sleep quality-related indicators such as PSQI, Sleep Quality VAS, MOS-SS, EORTC QLQ C30, etc. We have provided a PRISMA checklist, as detailed in **Supplementary Document 1**.

#### 2.2.2 Exclusion criteria

(1) Conferences, abstracts, reviews, case reports and synthesis literature; (2) duplicate publications; (3) non-RCTs; (4) studies with inaccessible full text or incomplete data; and (5) literature in languages other than English.

### 2.3 Statistical analysis

All retrieved literature was remitted to NoteExpress literature management software and duplicates were removed. Two researchers independently read the titles and abstracts of the articles. If they initially met the inclusion criteria, the full text was further read and screened according to the inclusion and exclusion criteria. In case of disagreements, a third researcher was consulted or discussions and negotiations were held within the research group to resolve the issue. In case of missing or unclear data, the authors of the articles were contacted personally by e-mail, etc. The two researchers followed the independent double-blind principle to extract the relevant information from the included literature. The main contents of the extraction included the first author, year of publication, sample size, tumor stage, intervention variables (intervention format, intervention type, weekly intervention frequency, total intervention duration, single intervention duration and intervention evaluation at different time points), outcome evaluation index (sleep evaluation tools such as PSQI, Sleep Quality VAS, MOS -SS, EORTC QLQ C30, etc.) and evaluation time.

### 2.4 Quality assessment

Quality assessment was performed by the two researchers independently using the Cochrane Handbook 5.1.0 evaluation criteria. In case of disagreement, the decision was discussed with a third researcher. The risk of bias assessment tool recommended by the Cochrane Handbook was used to evaluate the risk of bias in the included literature in seven areas: random sequence generation, allocation concealment, blinding of participants and implementers, blinding of outcome assessors, incomplete outcome data, selective reporting and other sources of bias. Each element was evaluated as “low risk of bias,” “unclear,” or “high risk of bias”.

### 2.5 Statistical analysis

RevMan 5.4.1 software was used for the statistical analysis of continuous variable data. Standard mean difference (SMD) effect scales and 95% confidence interval (CI) were selected for statistical purposes. The heterogeneity between the studies was examined using I^2^. If there was no or little heterogeneity between studies (P > 0.1, I^2^ < 50%), a fixed effects model was used for analysis, and if the heterogeneity was significant (P < 0.1, I^2^ > 50%), a random effects model was used for analysis. P < 0.05 indicated that the difference was significant.

## 3 Results

### 3.1 Literature search results

**Figure 1:**
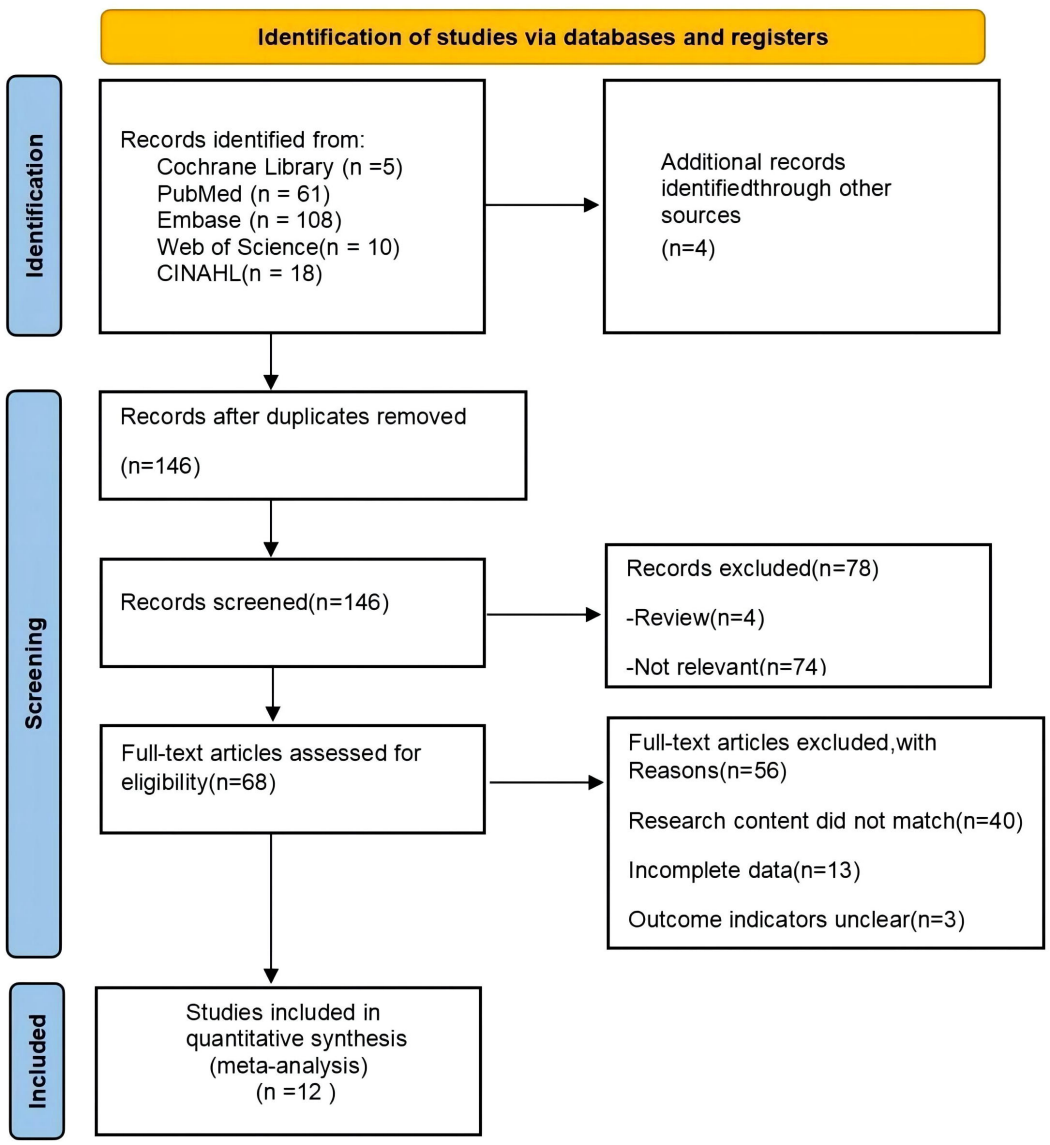
Flow chart of literature screening for inclusion.

A total of 202 documents were retrieved through the search strategy, PubMed(n=61),EMbase(n=108),the Cochrane Library(n=5),Web of Science(n=10),CINAHL(n=18),and 56 duplicates were removed in NoteExpress literature management software. A total of 78 documents were excluded by screening the titles and abstracts according to the inclusion and exclusion criteria. The reasons for exclusion were: irrelevant (n = 74); and review category (n = 4). Fifty-six publications were excluded by reading the full text, and the reasons for exclusion were as follows: study content did not match (n = 40); incomplete data (n = 13); and unclear outcome indicators (n = 3). Twelve RCTs were finally included (10–21). The literature screening process is shown in **Figure 1**.

### 3.2 Basic characteristics of included literature

**Table 1** shows the basic information of the included studies. Twelve RCTs were included (10–21) with a total sample size of 782, namely 393 in the yoga exercise group and 389 in the control group. The study population was breast cancer patients. The years of publication of the included studies were 2009–2019. The types of yoga included Hatha yoga (symmetrical positive yoga, emphasizing concentration on each movement, meditation techniques and awareness training), Tibetan yoga (emphasizing mind-body practice, controlled breathing and positive thinking techniques) and Integrated yoga (also known as comprehensive yoga, without prescribed techniques), mostly based on the principles of attention shifting and relaxation for mind-body training.

**Table 1:** General information of the included studies.

Author and year of publication	Age(Year)		Sample size (cases)	Tumour Staging	Therapy for tumors		Interventions		Cycle, frequency, time, form	Closing indicators	Evaluation time
	Intervention group	control group	Intervention group/control group		Intervention group	control group	Intervention group	control group			
Porter 2019	56.3 ± 11.6	59.4 ± 11.3	43/20	Phase IV	No restrictions	No restrictions	Orthomolecular Yoga	Active control group	8 weeks, 1 time per week, 120min per session, group	PSQI	Post-intervention and 1 month, 4 months post-intervention
Chaoul 2018	49.5 ± 9.8	49 ± 10.1	74/68/85	Phase II, III	Chemotherapy	hemotherapy	Tibetan Yoga	Routine care/stretching exercises	12 weeks, 1 time per week, 75~90min each time, individual	PSQI	Post-intervention and post-intervention 1 week, 3 months, and 6 months, 12 months
Rao 2017	48.9 ± 9.1	50.2 ± 9.2	45/46/56	Phase IV	No restrictions	No restrictions	Orthomolecular Yoga	Positive control group/stretching training	12 weeks, 2 times a week, 90min per session, 60min per session, individual	PSQI	Post-intervention
Ratcliff 2016	≥18	≥18	53/54/56	Phase 0~III	No restrictions	No restrictions	Integrated Yoga	Routine Care	6 weeks, 3 times a week, 60min each time, individual	PSQI	Post-intervention and 1 month, 3 months, 6 months after intervention
Yagli 2015	68.58 ± 6.17	68.88 ± 2.93	10/10	Phase I,II	Chemotherapy	hemotherapy	Yoga Training	Stretching training	8 weeks, 2 times a week, 60min each time, group	Sleep Quality VAS	Post-intervention
Danhauer 2015	50(29 - 83)	45(30–65)	22/18	Phase I,III	No restrictions	No restrictions	Integrated Yoga	Active control group	10 weeks, 1 time per week, 75min each time, individual	MOS-SS	Post-intervention
Chandwani 2010	51.39 ± 7.97	49.02 ± 9.96	30/31	Phase 0~III	radiation	radiation	Orthomolecular Yoga	Routine Care	6 weeks, 2 times a week, 60min each time, individual	PSQI	Post-intervention and post-intervention 1 week, 1 month, and 3 months
Carson 2009	53.9 ± 9.0	54.9 ± 6.2	17/20	Phase I,II	No restrictions	No restrictions	Orthomolecular Yoga	Routine Care	8 weeks, 1 time per week, 120min each time, group	Frequency of sleep disorders	Post-intervention, 1 month post-intervention
Vadiraja 2009	30-70	30-70	44/44	Phase II,III	No restrictions	No restrictions	Orthomolecular Yoga	Active control group	6 weeks, 3 times a week, 60min each time, individual	EORTC QOL C30- insomnia	Post-intervention
Lötzke 2016	51.0 ± 11.0	51.4 ± 11.1	45/47	Phase I-III	No restrictions	No restrictions	yoga intervention (YI)	physical exercise intervention (PEI)	12 weeks, once a week, 60min each time, individual	EORTC QOL C30	Post-intervention,3 months post-intervention
Pasyar2019	51.6 ± 10.46	51.8 ± 11.4	20/20	Phase 0-III	No reference	No reference	yoga exercise program plus the routine BCRL care.	Conventional treatment	8 weeks, 3 times a week (2 times, 1 time at home), group + individual	EORTC QLQ C30	4 weeks post-intervention, 8 weeks post-intervention
Jong2018	51 ± 8	51 ± 7.3	42/29	Phase I-III	No restrictions	No restrictions	Yoga+SC	Standard Care SC	12 weeks, once a week, 75min per session, team	EORTC QLQ C30	3 months, 6 months post-intervention

PSQI is Pittsburgh Sleep Quality Index; Sleep Quality VAS is Sleep Quality Visual Analogue Score; MOS-SS is MOS Sleep Scale; EORTC QOL C30-insomnia is Insomnia Dimension of European Quality of Life Scale; Routine care is routine care measures given; Active control group is health talk through social support group; Stretching training is simple stretching exercise.

The intervention in the exercise program consisted of three formats of yoga exercise intervention: group exercise, individual exercise and group + individual exercise. The frequency of intervention ranged from one to three times per week, with once per week being the most frequent. The intervention period ranged from 6 to 12 weeks, with 8 and 12 weeks being the most frequent. The duration of a single intervention ranged from 60 to 120 min, with a high concentration of 60 min. Only one study did not mention the duration of a single intervention.

### 3.3 Quality assessment

**Figure 2:**
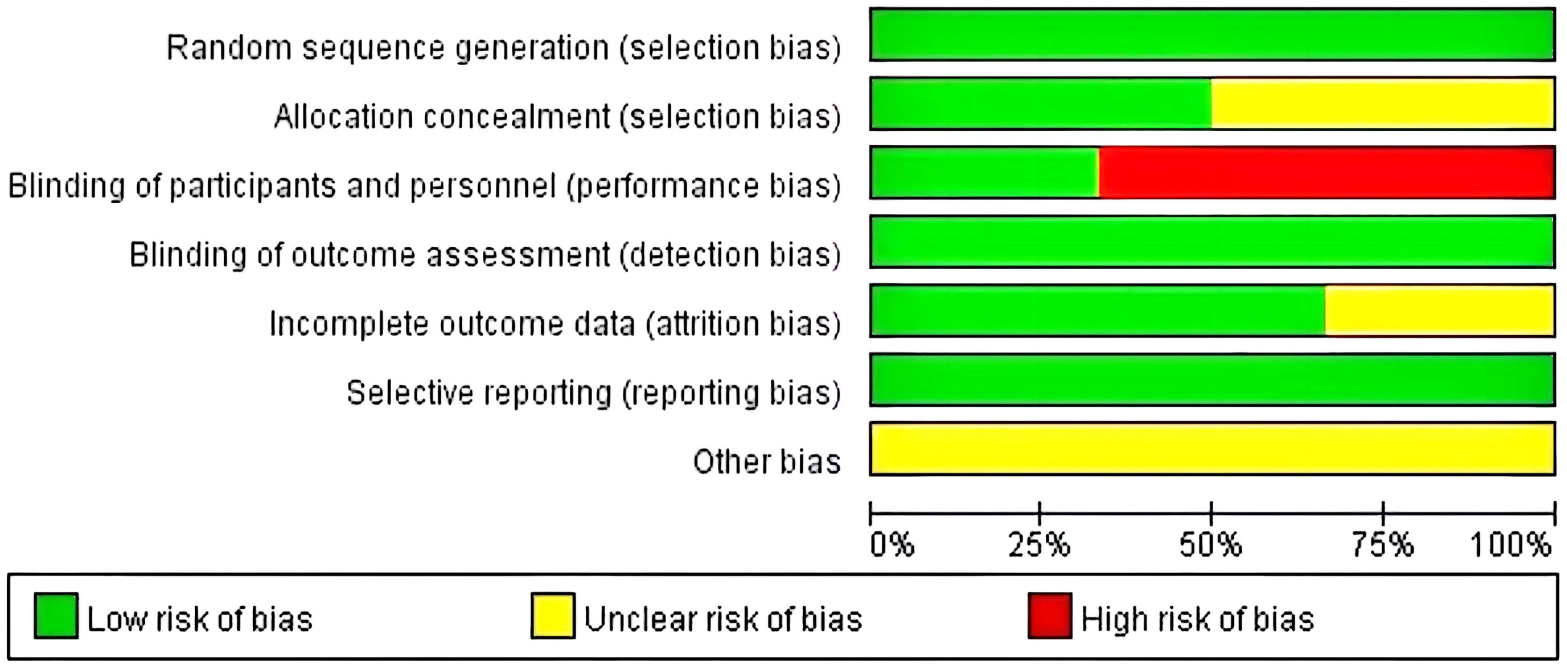
Overview of bias evaluation in the included literature.

**Figure 3:**
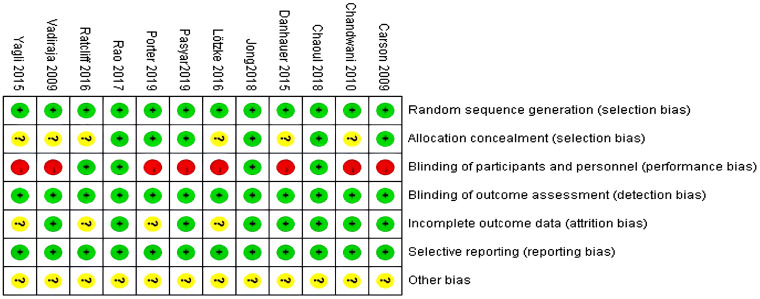
Risk of bias evaluation graph for the included literature.

The risk of bias assessment tool recommended by the Cochrane Collaboration was used for the evaluation. Among the 12 included studies, the methodological quality of all 12 was moderate bias, and the evaluation of the included studies using the Cochrane Systematic Assessment suggested that the average quality of the literature was Grade B. The quality assessment results of each study are shown in **Figures 2** and **3**.

### 3.4 Results of meta-analysis

#### 3.4.1 Overall test of intervention effects

**Figure 4:**
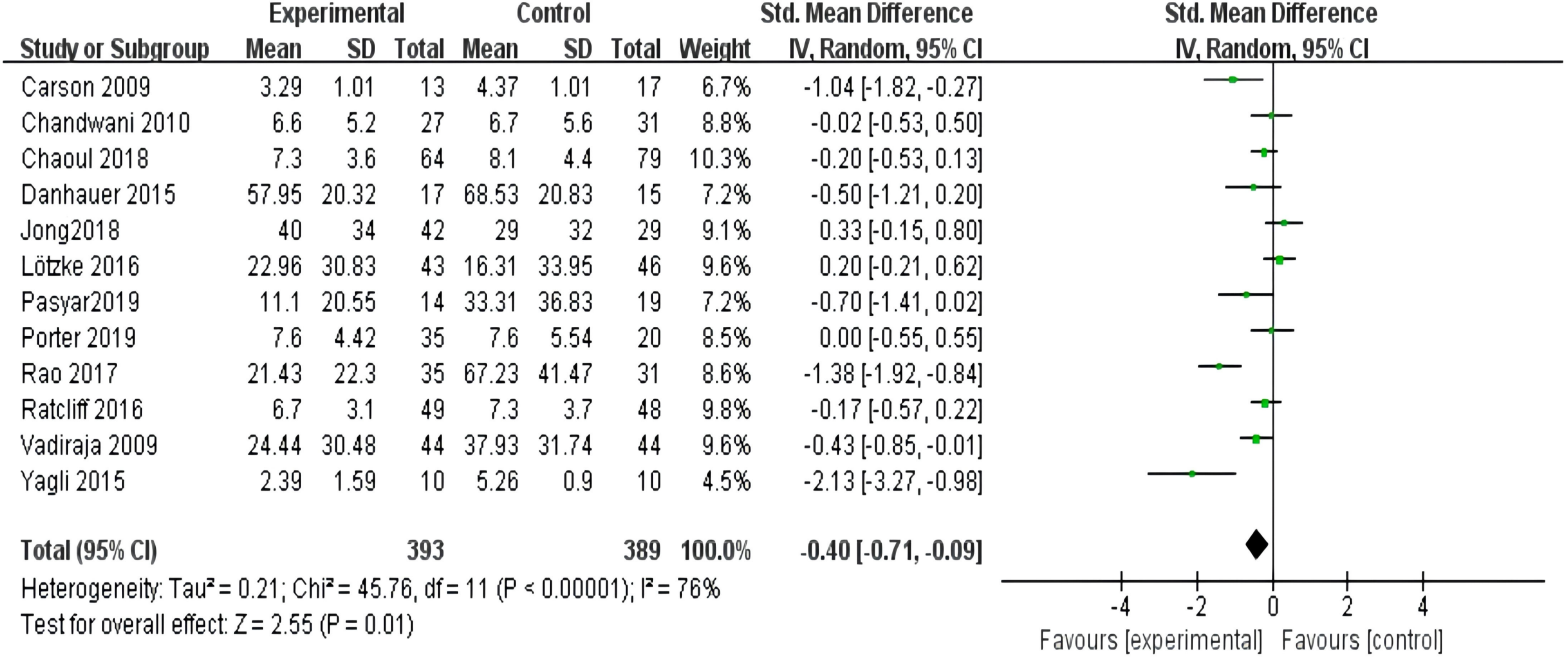
Forest plot of the overall effect of yoga intervention exercise to improve sleep quality in breast cancer.

An overall intervention effect size test on the entire sample of 12 selected papers (10–21) found that yoga exercise intervention had a good effect in improving sleep quality in breast cancer patients. The overall heterogeneity of the included literature was tested (I^2^ = 76%, p < 0.01) and the effect sizes were combined using a random effects model. The large degree of heterogeneity among multiple datasets in the meta-analysis suggests the possibility of multiple moderating variable factors affecting the overall effect size. **Figure 4** shows that the combined effect size was [SMD = -0.40 (P = 0.01) 95% CI: (-0.71, -0.09)], indicating that yoga exercise intervention achieved certain effects in improving sleep quality. P < 0.05, indicating that the combined effect size of multiple datasets was statistically significant.

#### 3.4.2 Subgroup analysis of moderating variables

Based on the heterogeneity of the overall effect size test, further subgroup analyses of the moderating variables were needed to explore the sources of heterogeneity. The effects of yoga exercise intervention on the quality of sleep in breast cancer patients may be influenced by the intervention variables, so this study set up subgroups to test each of the six variables of the exercise program: intervention format, intervention type, weekly intervention frequency, total intervention duration, single intervention duration and intervention evaluation at different time points.

##### 3.4.2.1 Meta-analysis of effects of different formats of yoga exercise intervention on sleep quality in breast cancer patients

**Figure 5:**
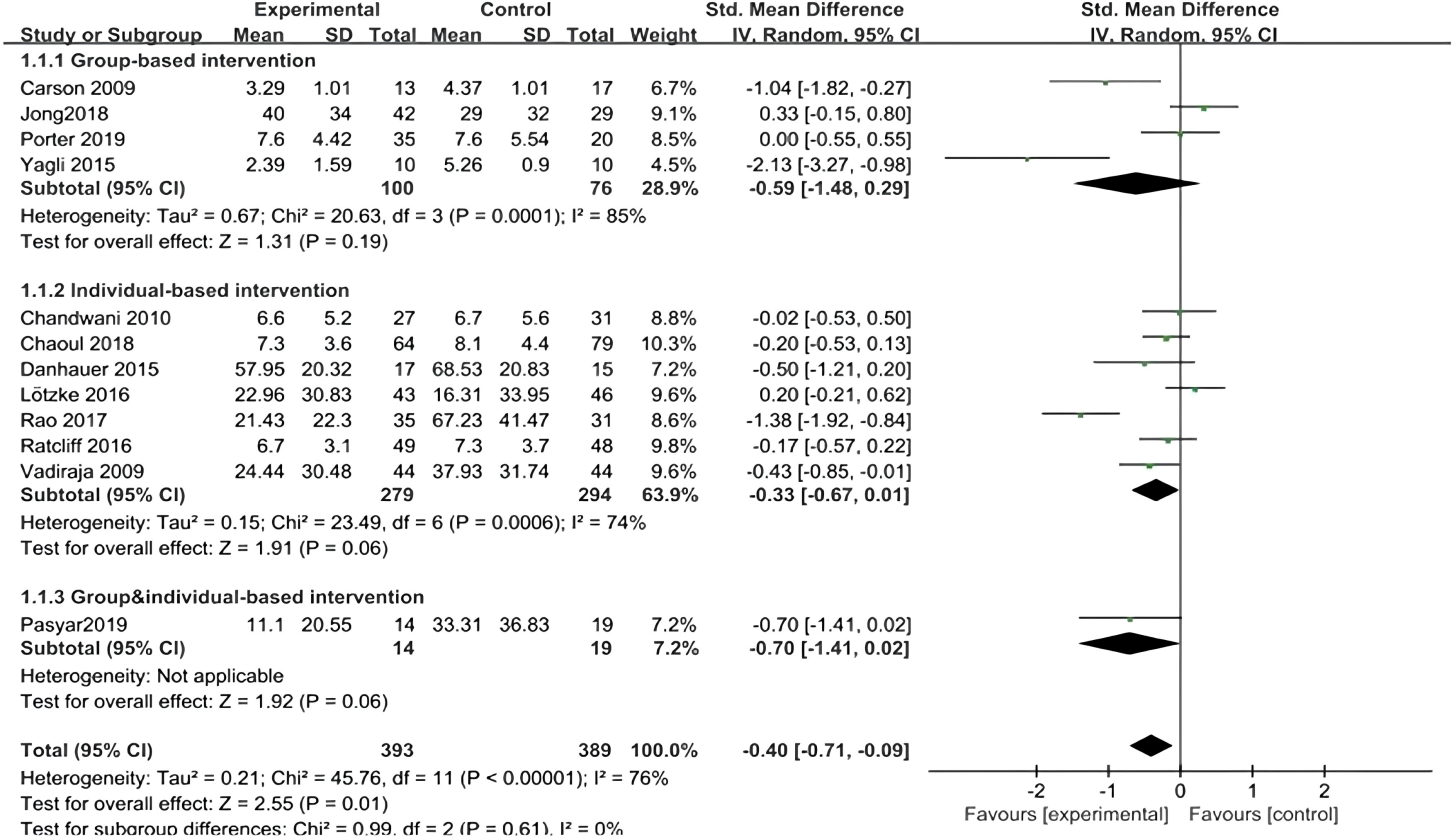
Forest plot of the effect of different forms of yoga interventions on improving sleep quality in breast cancer patients.

All twelve studies (10–21) reported the effects of different formats of yoga exercise intervention, and the heterogeneity test showed large heterogeneity (I^2^ = 76%, P < 0.001), so a random effects model was used; the results after combining the effect sizes showed a statistically significant difference in sleep effects between the three groups at the end of the intervention [SMD = -0.40, 95% CI: (-0.71, - 0.09), P = 0.01]. However, there was no statistically significant difference in the intervention effect of the group form (P=0.19), and there was also no statistically significant difference in the intervention effect of the individual form (P=0.06). The difference in intervention effect between the team and individual form (P=0.06) was also not statistically significant, as shown in **Figure 5**.

##### 3.4.2.2 Meta-analysis of effects of different types of yoga exercise intervention on sleep quality in breast cancer patients

**Figure 6:**
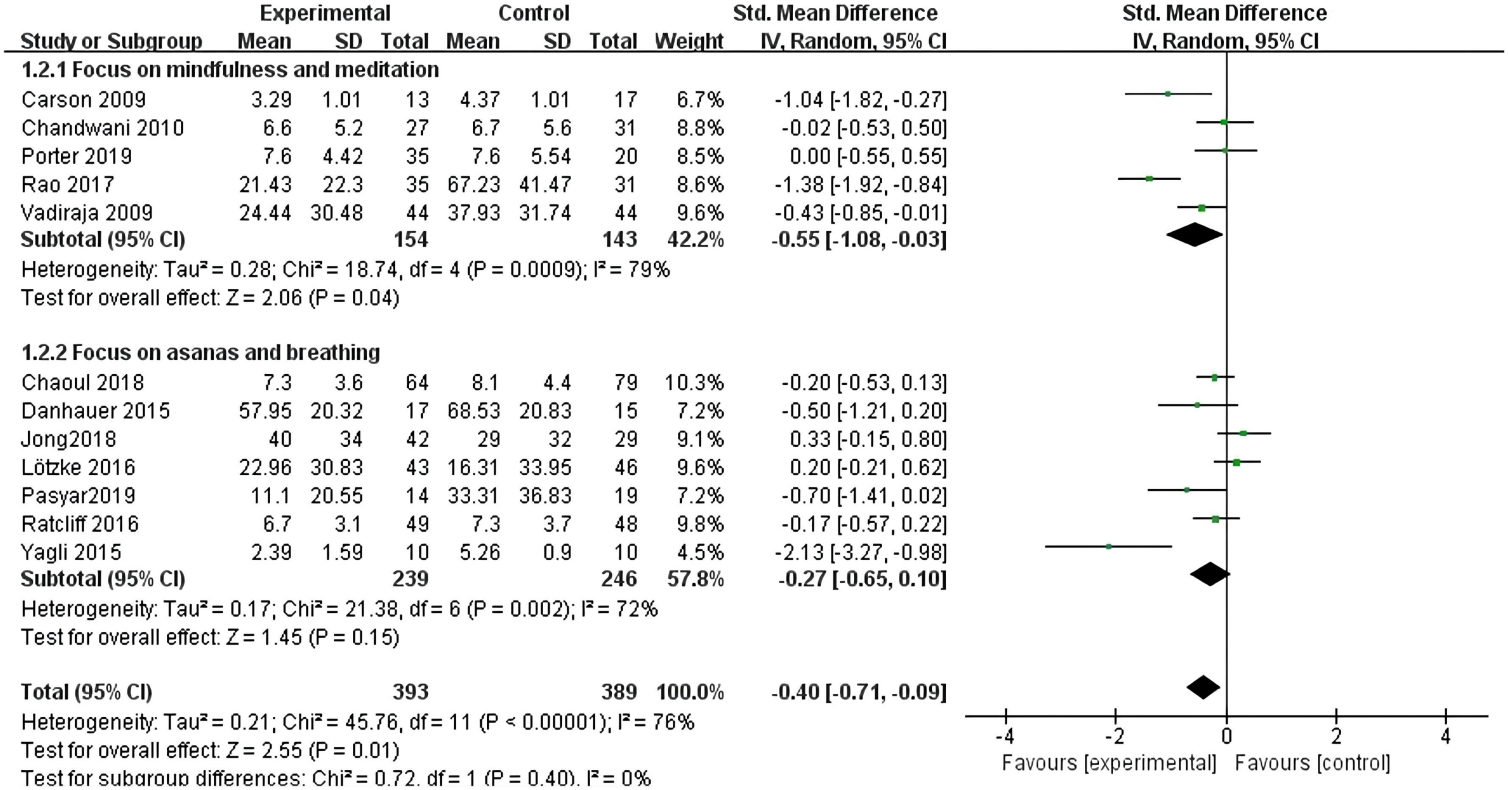
Forest plot of the effect of different types of yoga interventions on improving sleep quality in breast cancer patients.

All twelve studies (10–21) reported the effects of different types of yoga exercise intervention. This study grouped yoga focusing on mindfulness and meditation into one category, and yoga focusing on asana and breathing into another. The results of the heterogeneity test showed large heterogeneity (I^2^ = 76%, P < 0.001), so a random effects model was used; the results after combining the effect sizes showed that yoga with a focus on meditation was better than conventional care [SMD = -0.55, 95% CI: (-1.08, -0.03), P = 0.04]. However, the difference in the effects of intervention with a focus on asana and breathing was not statistically significant (P = 0.15) (see **Figure 6**).

##### 3.4.2.3 Meta-analysis of effects of different weekly intervention frequencies on sleep quality in breast cancer patients

**Figure 7:**
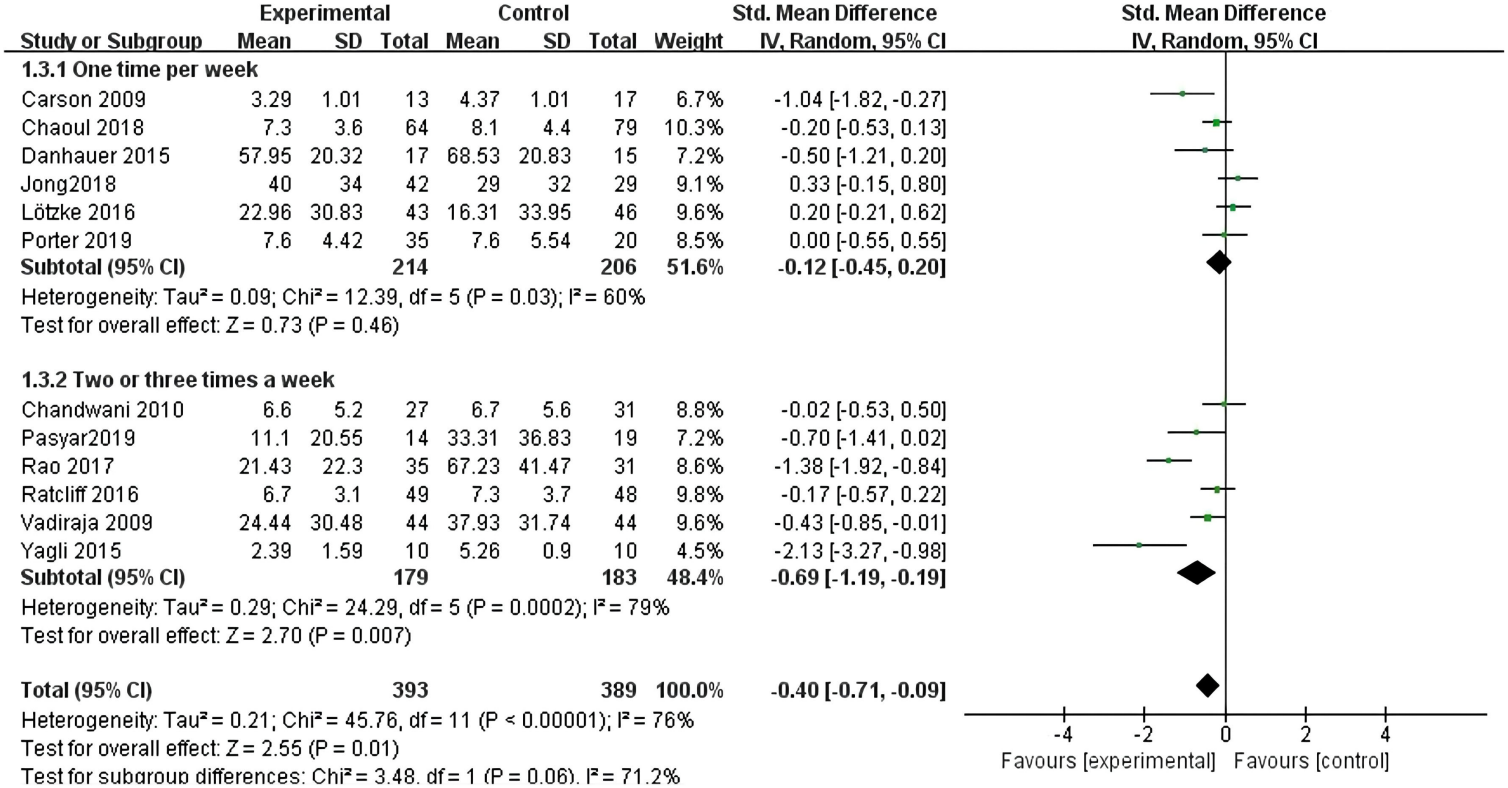
Forest plot of the effect of different frequencies of yoga interventions per week on improving sleep quality in breast cancer patients.

All twelve studies (10–21) reported the effects of yoga exercise intervention with different weekly frequencies. The results of the heterogeneity test showed large heterogeneity (I^2^ = 76%, P < 0.001), so a random effects model was used; the results after combining the effect sizes showed a statistically significant difference in sleep effects between the two groups with two or three weekly intervention times [SMD = -0.69, 95% CI: (-1.19, -0.19), P = 0.007], but for sleep effects between the two groups with one weekly intervention, the difference was not statistically significant (P = 0.46) (see **Figure 7**).

##### 3.4.2.4 Meta-analysis of effects of different total intervention durations on sleep quality in breast cancer patients

**Figure 8:**
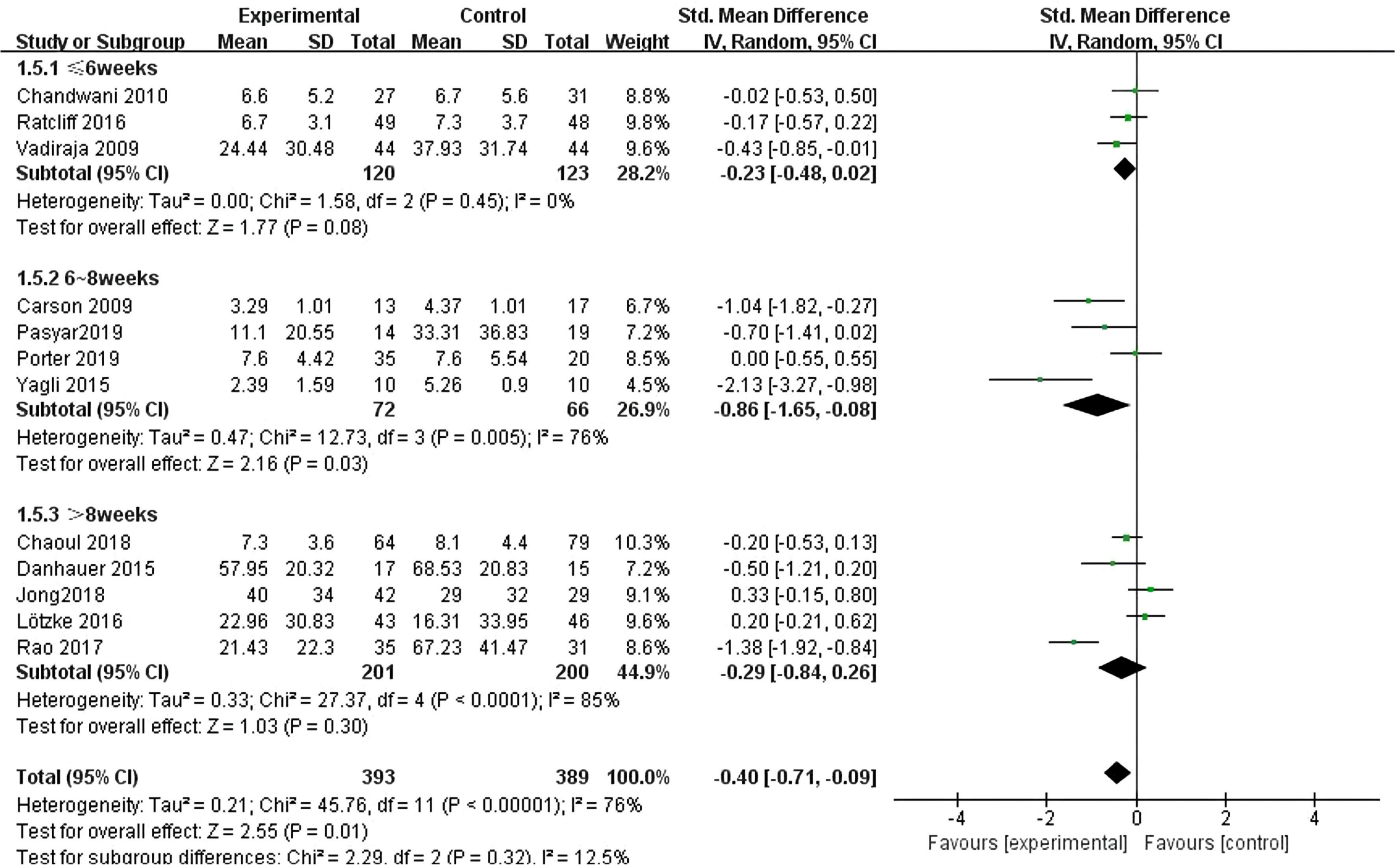
Forest plot of the effect of yoga interventions of different total duration on improving sleep quality in breast cancer patients.

All twelve studies (10–21) reported the effects of yoga exercise intervention with different total intervention durations. The results of the heterogeneity test showed large heterogeneity (I^2^ = 76%, P < 0.001), so a random effects model was used; the results after combining the effect sizes showed a statistically significant difference in sleep effects for intervention periods of 6–8 weeks [SMD = -0.86, 95% CI: (-1.65, -0.08), P = 0.03]. However, there was no statistically significant difference in sleep effects between intervention cycles ≤ 6 weeks; The difference in sleep effects greater than 8 weeks was also not statistically significant (see **Figure 8**).

##### 3.4.2.5 Meta-analysis of effects of different single intervention durations on sleep quality in breast cancer patients

**Figure 9:**
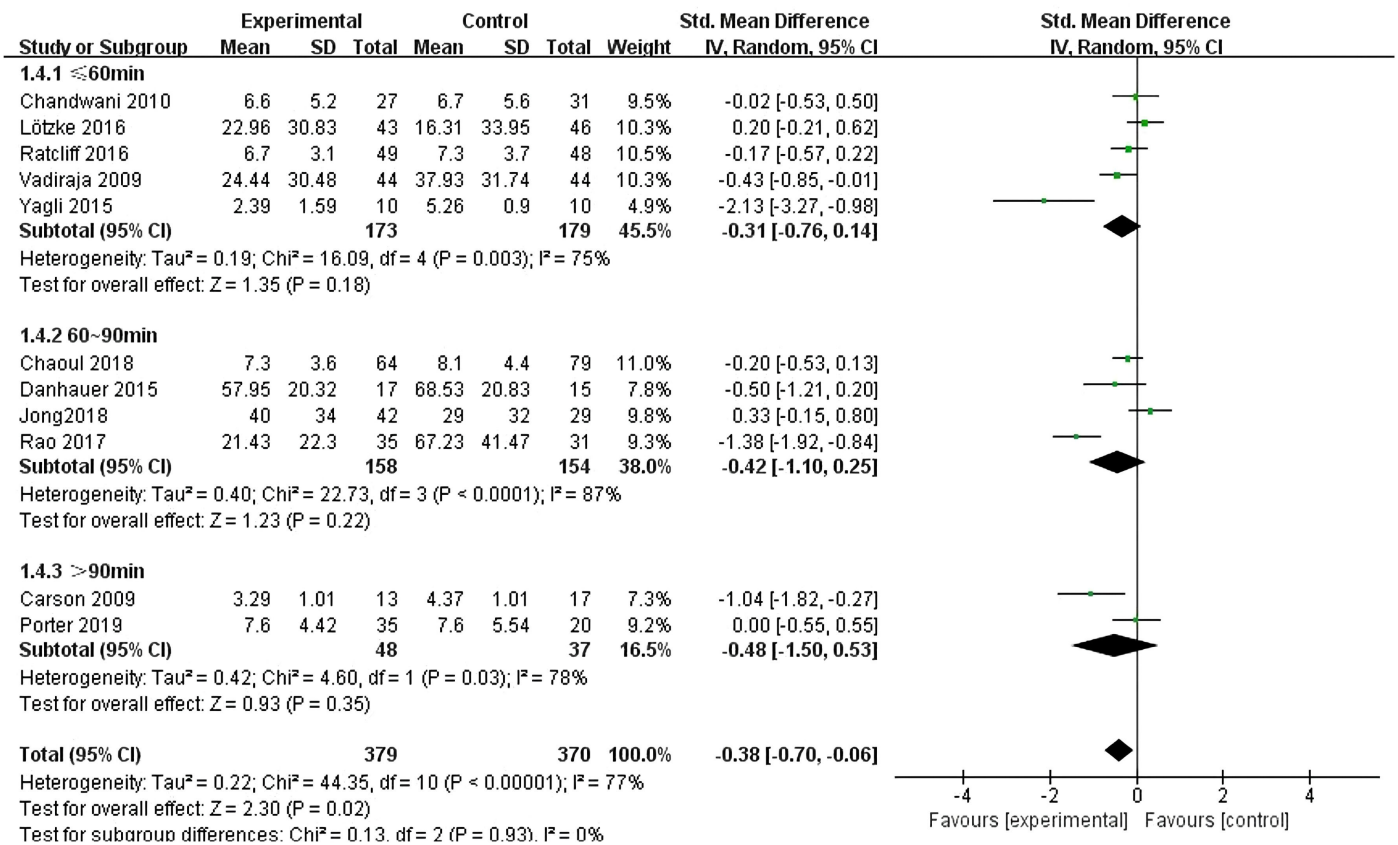
Forest plot of the effect of different single session yoga intervention lengths on improving sleep quality in breast cancer patients.

Eleven studies (10–20) reported the effects of yoga exercise intervention with different total intervention durations. The results of the heterogeneity test showed large heterogeneity (I^2^ = 77%, P < 0.001), so a random effects model was used; the results after combining the effect sizes showed a statistically significant difference in sleep effects between the three groups at the end of the intervention [SMD = -0.38, 95% CI: (- 0.70, -0.06), P =0.02]. Further subgroup analysis showed that the intervention effect increased with the duration of a single intervention (-0.31, -0.42, -0.48), and meta regression analysis showed that the intervention time had a significant moderating effect, as shown in **Figure 9**.

##### 3.4.2.6 Meta-analysis of effects of evaluation of intervention at different time points on sleep quality in breast cancer patients

**Figure 10:**
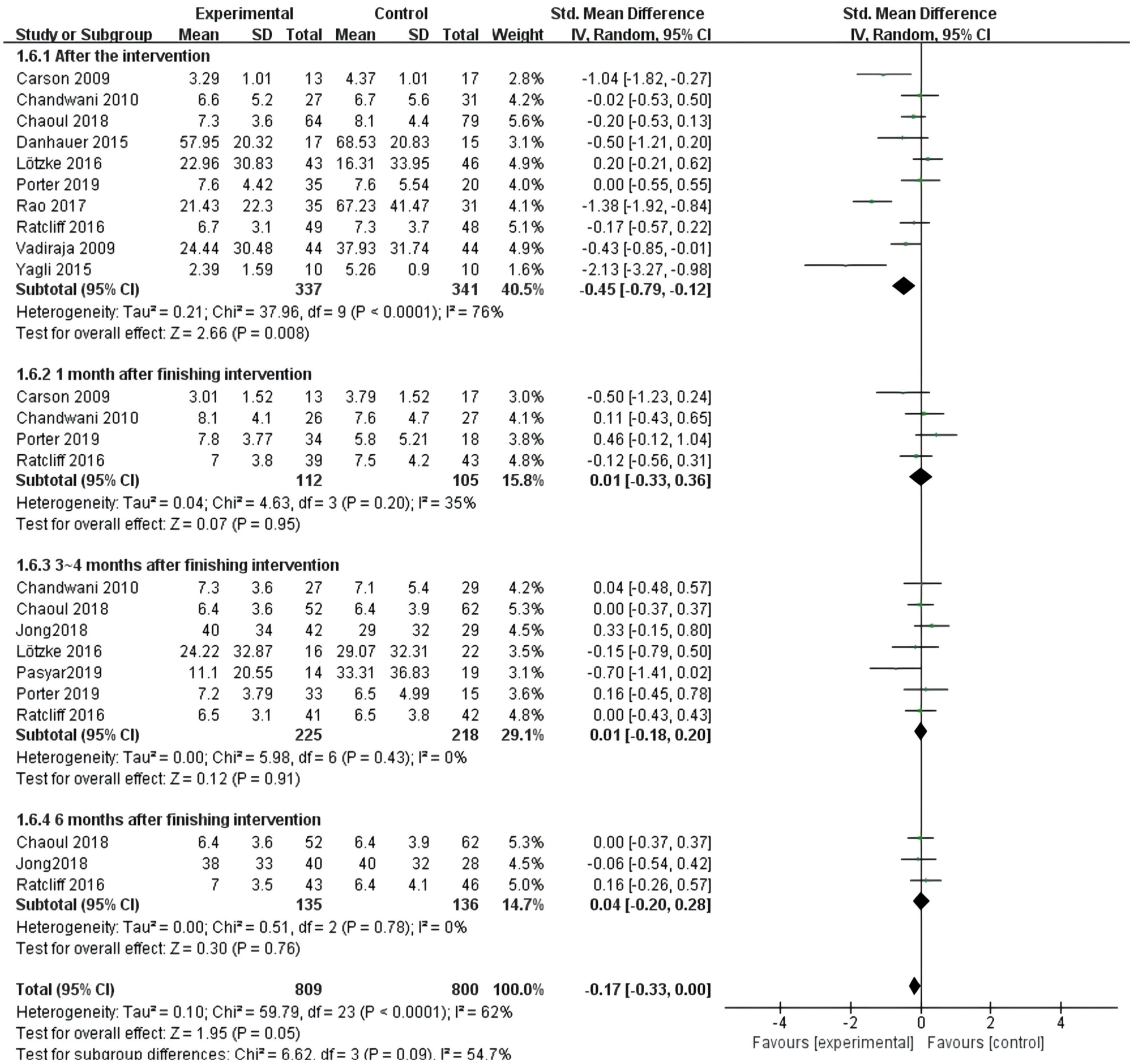
Forest plot of the effect of yoga intervention evaluation at different time points on improving sleep quality in breast cancer patients.

All twelve studies (10–21) reported the effects of yoga exercise intervention with evaluation at different time points. The heterogeneity test showed large heterogeneity (I^2^ = 62%, P < 0.001), so a random effects model was used; the results after combining the effect sizes showed a statistically significant difference in sleep effects between the four groups at the end of the intervention [SMD = -0.17, 95% CI: (-0.33, 0.00), P =0.05]. Among them, the difference in sleep effects evaluated at the end of the intervention was statistically significant [SMD = -0.45, 95% CI: (-0.79, -0.12), P =0.008]. However, there was no statistically significant difference in intervention effect after one month of intervention (P=0.95), and there was no statistically significant difference in intervention effect after 3-4 months (P=0.91). Similarly, there was no statistically significant difference in intervention effect evaluated after 6 months (P=0.76) (see **Figure 10**).

### 3.5 Sensitivity analysis

Sensitivity analysis is a method used to evaluate whether the results of a meta-analysis or systematic evaluation are stable and reliable. Sensitivity analysis was performed on the 12 included papers, mainly by excluding papers one by one. The combined results and interstudy heterogeneity did not change significantly after excluding any of the papers, indicating that the results of this meta-analysis are reliable. We further conducted sensitivity analysis using Stata 12.0 software, indicating that the system evaluation results are stable and reliable (see **Supplementary Material 2**).

### 3.6 Publication bias analysis

**Figure 11:**
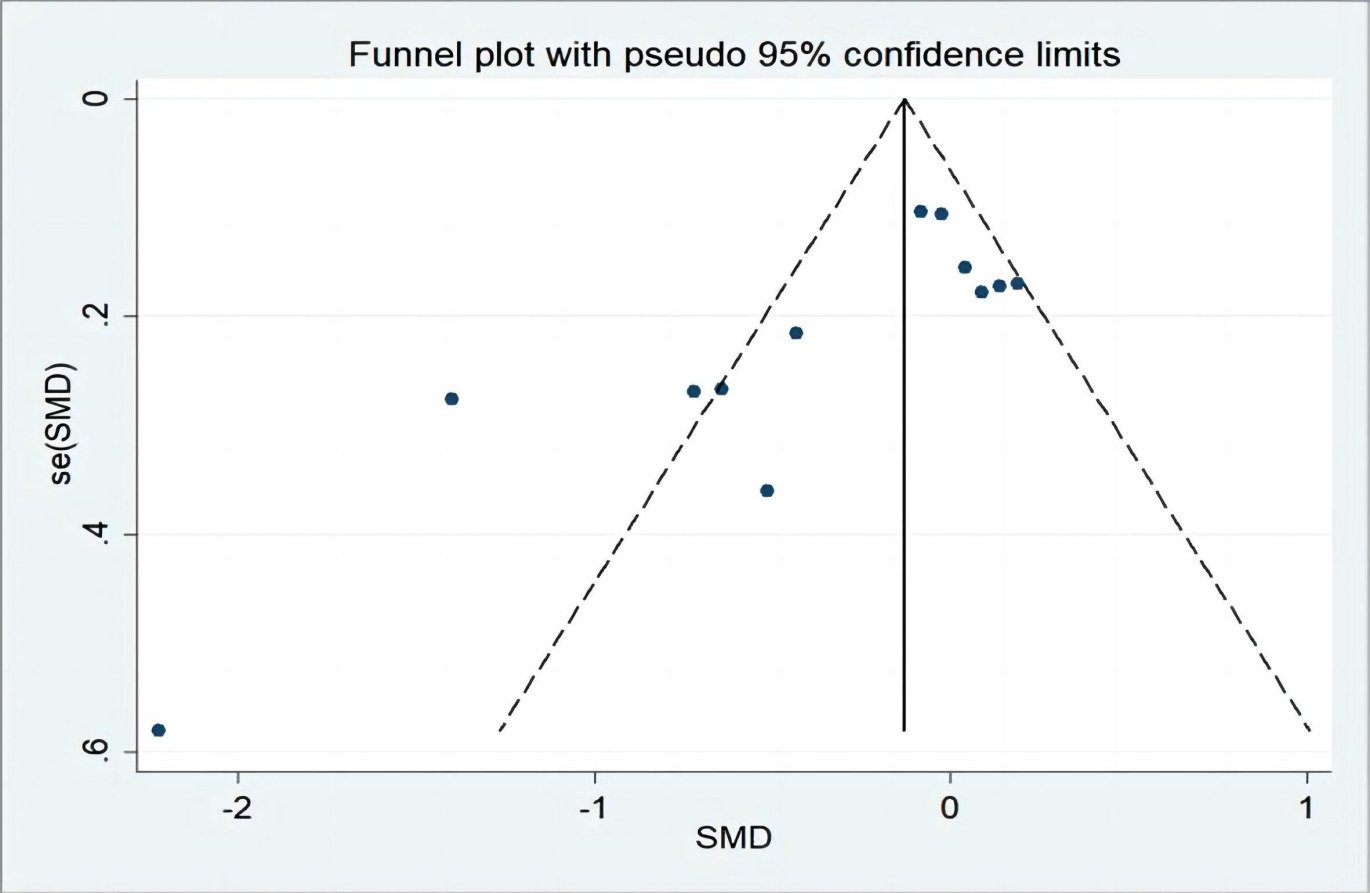
Publication of biased funnel plots.

**Figure 12:**
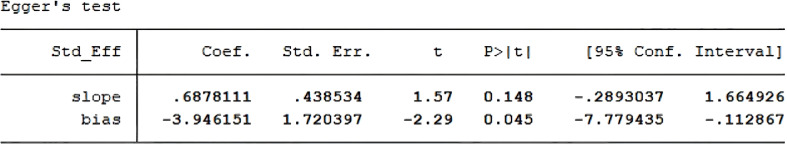
Funnel plot of Egger’s method to assess publication bias.

Twelve studies were included, allowing for a test of publication bias. Publication bias analysis was performed using a plotted funnel plot, and the results showed (see **Figure 11**) that four papers deviated by a certain degree from the rest of the literature, indicating mild heterogeneity. Asymmetry was observed in the funnel plot, confirming that there was some publication bias in the included literature, which may be related to phenomena such as the preference of scholars for positive results in the published literature. In addition, we also used the Egger method to evaluate whether there is publication bias. In this case, the Egger test (see **Figure 12**) showed a result of P=0.045<0.05, indicating mild publication bias. We consider that these biases may be related to different stages of breast cancer. The results of the meta-analysis of Maqbali et al. (22) show that the highest proportion of sleep disorders is 70.8%, which indicates that sleep disorders may be related to tumor stages, which may cause yoga to have different sleep effects on breast cancer patients with different tumor stages. The literature included in our meta-analysis failed to provide detailed breast cancer tumor staging data, and further subgroup analysis from breast cancer tumor staging was impossible, which affected the reliability of the results. At the same time, this may also be one of the reasons for the high heterogeneity of the article, and we expect larger sample, multicenter studies to further confirm in the future.

## 4 Discussion

Yoga is an ancient Eastern fitness practice that originated in India. Traditionally, Indian yoga involves multiple domains, including ethical concepts, physical postures and spiritual practices, in order to achieve the unity of mind and body, mental health and self-awareness (23). A yoga trial involving breast cancer survivors with chronic fatigue showed that yoga was safe and effective in improving fatigue severity, depression and sleep quality (24). There is growing evidence to support the use of yoga to improve the side effects of cancer treatment such as fatigue, anxiety and sleep disorders (25–27). The clinical guidelines of the Society for Integrative Oncology recommends yoga for common conditions such as anxiety and mood disorders among women with breast cancer (28), and it has also been endorsed by the American Society of Clinical Oncology (29). The authors systematically reviewed studies on the effects of yoga exercise intervention in order to improve sleep quality in breast cancer patients.

Our meta-analysis included 12 studies involving 782 experimental subjects. The overall intervention effect size test showed that yoga exercise intervention had good effects in improving sleep quality in breast cancer patients. Carson et al. (10) studied the adoption of positive yoga exercise intervention in the format of a group intervention of 120 min once a week, and the results after 1 month showed that it helped to reduce the frequency of sleep disorders. Vadiraja et al. (11) studied positive yoga exercise intervention in the format of an individual intervention of 60 min three times a week, and the results after 8 weeks of intervention showed that it helped to improve the sleep score of EORTC QOL C30; Yagli et al. (14) studied positive yoga training in the format of 60 min two times a week, and the results after 8 weeks of intervention showed that it helped to improve the sleep quality scores; Rao et al. (17) studied positive yoga training in the format of 60 or 80 min two times a week, and the results after 12 weeks of intervention showed that it helped to reduce the PSQI scores. More recently, Mustian et al. (30) published the first and only multicenter phase III RCT that assessed the effects of yoga on insomnia and sleep quality impairment through validated patient-reported measures and objective activity recording measures. This is the most definitive clinical trial to date that shows that yoga is effective in improving insomnia and sleep quality impairment compared to usual care control conditions. The yoga participants reported significant moderate to substantial improvements in insomnia and sleep quality impairment outcomes, as well as significant improvements in the objective activity recording assessments of sleep outcomes (including waking after sleep and sleep efficiency). The yoga participants also showed a significant 21% reduction in sleep medication use, while the control participants showed a 5% increase in sleep medication use. Adherence to yoga reached 80% and there were no adverse events associated with the study. The mechanism by which yoga enhances sleep in breast cancer patients may be because it is a mode of positive physical activity, and therefore improves sleep disorders by increasing melatonin levels, reducing excessive wakefulness and addressing stress-related cardiac and respiratory abnormalities (31).

The meta-analysis of yoga exercise intervention types showed that positive yoga + meditation exercise intervention had a significant effect on the improvement of sleep quality. Yoga combines posture, breathing and meditation, focuses not only on the practice of posture but also on mental practice, and emphasizes not only physical but also mental relaxation, allowing participants to better regulate their emotions (32, 33). Reich et al. (34) showed that that meditation guides patients to focus on their emotions and feelings, and encourages them to accept their environment with a peaceful mind, thus promoting sleep.

The meta-analysis of yoga exercise intervention frequency showed that intervention two or three times per week was more effective in improving sleep, but the credibility of this finding requires further validation in more multicenter large-sample studies due to the large heterogeneity. However, due to the lack of studies with more than three intervention times per week, it is not yet possible to prove that the higher the intervention frequency, the better the intervention effects, so it is difficult to derive the optimal frequency of yoga practice. Thus, as the current evidence only recommends practicing yoga exercise intervention two to three times per week, participants are not encouraged to increase their intervention frequency to improve their sleep quality.

The meta-analysis of the evaluation of yoga exercise intervention at different time points showed that sleep improvement in the yoga exercise intervention group was significant at the end of the intervention, but the differences were not statistically significant at 1 month, 3–4 months and 6 months after intervention, indicating that the short-term effects of the intervention were significant, but the long-term effects were not significant. However, due to the large heterogeneity, the credibility of this conclusion needs to be further verified by more multicenter large-sample studies.

The meta-analysis of yoga exercise intervention format showed that the different formats of yoga exercise intervention such as group format, individual format and group + individual format did not show statistical differences in the effects of sleep quality in breast cancer patients. A meta-analysis of yoga single session length showed that the three different intervention lengths of ≤ 60 min, 60–90 min and > 90 min did not show statistical differences.

Limitations and shortcomings: (1) this study only included English literature, which may have generated some selective bias; (2) The small sample size included in the study will affect the reliability of the research results, and its conclusions need to be treated with caution.(3)the included breast cancer patients had different tumor stages, so their degree of sleep disorders may also have been different, which could have easily affected the stability and reliability of the results; (4) In the included studies, the mismatch of age, gender, performance status and treatment status between the yoga group and the control group may lead to different sleep effects of yoga on breast cancer patients, leading to risk bias of meta analysis. Unfortunately, the literature included in our meta-analysis did not provide detailed data on age, gender, presentation status, and treatment status, making it impossible to conduct further subgroup analysis from these confounding variables, which affected the reliability of the results and may also be one of the reasons for the high heterogeneity of the article.(5)the outcome indicators of sleep quality are subjective sleep quality scores, and there is a lack of objective sleep measurement data such as sleep time and sleep frequency, which may have generated some bias; (6)Although meta-analysis shows that performing yoga interventions twice or three times a week can help improve sleep outcomes, the lack of research on intervening more than three times a week does not yet determine the optimal frequency of yoga practice and does not encourage participants to increase their frequency of extracurricular exercises to improve sleep quality. Additionally, the heterogeneity of the research also affects the stability of the results.(7)although high-quality literature was included and stringent inclusion criteria were followed, large heterogeneity was still found, and heterogeneity still existed when the subgroup analysis was performed, which affected the reliability of the results and may have caused some bias. Therefore, the conclusions of this meta-analysis need to be interpreted with caution, and further validation by multicenter large-sample prospective cohort studies is needed in the future.

## 5 Conclusion

The results of this meta-analysis show that yoga can effectively improve the sleep quality of breast cancer patients, and the most significant effects occur after positive yoga + meditation exercise intervention two or three times per week. Health professionals and patients should pay more attention to the effects of yoga exercise intervention on the sleep quality of breast cancer patients, and health professionals should guide breast cancer patients to perform reasonable yoga exercise to improve their sleep quality.

## Data availability statement

The raw data supporting the conclusions of this article will be made available by the authors, without undue reservation.

## Author contributions

JZZ, XC, XXZ, HZ, HC and YW are the guarantor of the manuscript and take responsibility for the content of this manuscript. XC, JZZ, XXZ and CW contributed to the design of the study. HMC, JRZ and HL were involved in the data analysis. CW and RC contributed to the acquisition of primary data. JZ, XC and ZZZ wrote the initial draft of the manuscript. JZ and YW contributed significantly to the revision of the manuscript. All authors contributed to the article and approved the submitted version.
