# Supplementary material for: Meta-analysis of effects of yoga exercise intervention on sleep quality in breast cancer patients
Source: Front Oncol. 2023 Jun 30;13:1146433. doi: 10.3389/fonc.2023.1146433 (PMC10348890; doi:10.3389/fonc.2023.1146433)
Supplement: Supplementary file 1 [file Table_1.docx]

| **Section and Topic** | **Item #** | **Checklist item** | **Location where item is reported** |
| --- | --- | --- | --- |
| **TITLE** | | |  |
| Title | 1 | The report is identified as a meta-analysis | Page 1 |
| **ABSTRACT** | | |  |
| Abstract | 2 | To systematically evaluate and test the effects of yoga exercise intervention programs on sleep quality in breast cancer patients by meta-analysis. | Page 2 |
| **INTRODUCTION** | | |  |
| Rationale | 3 | In recent years, more and more studies have begun to focus on the effects of yoga exercise intervention programs on sleep quality in breast cancer patients,Sleep disorders can impar the immune systems, cognitive abilities and daily functions of breast cancer patients, affect their emotions, as well as being closely related to tumor development and progression.It was recently suggested that yoga practice can improve overall sleep efficiency and total sleep time. The beneficial effects of yoga exercise on sleep are being increasingly recognized. | Page 2-3 |
| Objectives | 4 | The purpose of this study is to conduct a meta-analysis of the effects of yoga exercise intervention across different intervention variables on the sleep quality of breast cancer patients in order to provide the best evidence-based foundation for the effective improvement of patients' sleep. | Page 3 |
| **METHODS** | | |  |
| Eligibility criteria | 5 | Inclusion criteria: (1) subjects: patients aged ≥ 18 years with a pathological diagnosis of breast cancer; (2) intervention: yoga exercise intervention in the experimental group and unlimited care measures in the control group; (3) study type: RCT; (4) outcome indicators: sleep quality-related indicators such as PSQI, Sleep Quality VAS, MOS-SS, EORTC QLQ C30, etc. | Page 4 |
| Information sources | 6 | A comprehensive and systematic search was performed using the web databases of PubMed（ Date of inception 1940s - June 8, 2022）、Embase（Date of inception 1960 – June 8, 2022）、Cochrane Library（Date of inception 1999 – June 8, 2022）、Web of Science（Date of inception 1975 – June 8, 2022）and CINAHL（Date of inception 1982 – June 8, 2022）. | Page 3 |
| Search strategy | 7 | The search terms included free words as well as MeSH subject headings. The English terms used to search the literature were as follows: Breast Neoplasm, Breast Tumor, Breast Cancer, Mammary Cancer, Malignant Neoplasm of Breast, Human Mammary Neoplasm, Breast Carcinoma;yoga, yogic, asana, pranayama, yoga exercise; sleep quality, sleep. | Page 3 |
| Selection process | 8 | The quality of the articles was assessed by two researchers, and any disagreements were resolved through discussion until a consensus was reached or after consultation with the third author. | Page 4 |
| Data collection process | 9 | The three authors independently extracted the necessary data, and any disagreements were resolved through discussion until a consensus was reached or after consultation with the third author. The extraction of the relevant data from the included articles was then performed. | Page 4 |
| Data items | 10a | The extracted data included the first author, year of publication, sample size, tumor stage, intervention variables (intervention format, intervention type, weekly intervention frequency, total intervention duration, single intervention duration and intervention evaluation at different time points), outcome evaluation index (sleep evaluation tools such as PSQI, Sleep Quality VAS, MOS -SS, EORTC QLQ C30, etc.) and evaluation time. | Page 4 |
|  | 10b | Table 1 Basic Characteristics of Included Articles | Table 1 |
| Study risk of bias assessment | 11 | Quality assessment was performed by the two researchers independently using the Cochrane Handbook 5.1.0 evaluation criteria. In case of disagreement, the decision was discussed with a third researcher. The risk of bias assessment tool recommended by the Cochrane Handbook was used to evaluate the risk of bias in the included literature in seven areas: random sequence generation, allocation concealment, blinding of participants and implementers, blinding of outcome assessors, incomplete outcome data, selective reporting and other sources of bias. Each element was evaluated as "low risk of bias," "unclear," or "high risk of bias". | Page 4-5 |
| Effect measures | 12 | RevMan 5.4.1 software was used for the statistical analysis of continuous variable data. Standard mean difference (SMD) effect scales and 95% confidence interval (CI) were selected for statistical purposes. The heterogeneity between the studies was examined using I^2^. If there was no or little heterogeneity between studies (P > 0.1, I^2^ < 50%), a fixed effects model was used for analysis, and if the heterogeneity was significant (P < 0.1, I^2^ > 50%), a random effects model was used for analysis. P < 0.05 indicated that the difference was significant. | Page 5 |
| Synthesis methods | 13a | Table 1 Basic Characteristics of Included Articles | Table 1 |
|  | 13b | For the included articles, RevMan 5.4.1 software provided by Cochrane was used for the meta-analysis of all statistical analyses. | Page 5 |
|  | 13c | The incidence of PSQI, Sleep Quality VAS, MOS-SS, EORTC QLQ C30, etc in the breast cancer patients group and control group were used as outcome indicators. MD and OR values were used for effect evaluation, and 95% CI was calculated; the heterogeneity of the articles was evaluated through I^2^. If the included articles had no statistical heterogeneity (P > 0.01, I^2^ < 50%), the fixed effect model was used; if the included articles had statistical heterogeneity (P < 0.01,I^2^ > 50%), the random effect model was used, and sensitivity analysis or subgroup analysis was performed to understand the source of the heterogeneity. | Page 4-5 |
| Reporting bias assessment | 14 | Publication bias analysis was performed using a plotted funnel plot and Egger's method, and the results showed that four papers deviated by a certain degree from the rest of the literature, indicating mild heterogeneity. | Page 8 |
| Certainty assessment | 15 | MD values were used for the effect evaluation of sleep quality, and 95% CI was calculated. | Page 5 |
| **RESULTS** | | |  |
| Study selection | 16a | Fig. 1 Process of Inclusion of Articles in Meta-analysis | Fig 1 |
| Study characteristics | 17 | Cite each included study and present its characteristics. | Table 1 |
| Risk of bias in studies | 18 | Present assessments of risk of bias for each included study. | Fig 2,3 |
| Results of individual studies | 19 | Yoga exercise intervention had a good effect in improving sleep quality in breast cancer patients，and the combined effect size was [SMD = -0.40 (P = 0.01) 95% CI: (-0.71, -0.09)]. | Page 5-8  Fig 5-10 |
| Results of syntheses | 20a | For each synthesis, briefly summarise the characteristics and risk of bias among contributing studies. | Page 5-8 |
|  | 20b | Present results of all statistical syntheses conducted. If meta-analysis was done, present for each the summary estimate and its precision (e.g. confidence/credible interval) and measures of statistical heterogeneity. If comparing groups, describe the direction of the effect. | Page 5-8 |
|  | 20c | Present results of all investigations of possible causes of heterogeneity among study results. | Page 5-8 |
|  | 20d | Present results of all sensitivity analyses conducted to assess the robustness of the synthesized results. | Page 5-8 |
| Reporting biases | 21 | The presence of publication bias was assessed using Egger's method (Fig 12). | Fig 8 |
| Certainty of evidence | 22 | Present assessments of certainty (or confidence) in the body of evidence for each outcome assessed. | Page 5-8 |
| **DISCUSSION** | | |  |
| Discussion | 23a | Provide a general interpretation of the results in the context of other evidence. | Page 9-10 |
|  | 23b | Discuss any limitations of the evidence included in the review. | Page 9-10 |
|  | 23c | Discuss any limitations of the review processes used. | Page 9-10 |
|  | 23d | Discuss implications of the results for practice, policy, and future research. | Page 9-10 |
| **OTHER INFORMATION** | | |  |
| Registration and protocol | 24a | Not registered yet. |  |
|  | 24b | Indicate where the review protocol can be accessed, or state that a protocol was not prepared. | Page 11 |
|  | 24c | Describe and explain any amendments to information provided at registration or in the protocol. | Page 11 |
| Support | 25 | Describe sources of financial or non-financial support for the review, and the role of the funders or sponsors in the review. | Page 11 |
| Competing interests | 26 | Declare any competing interests of review authors. | Page 11 |

*From:*  Page MJ, McKenzie JE, Bossuyt PM, Boutron I, Hoffmann TC, Mulrow CD, et al. The PRISMA 2020 statement: an updated guideline for reporting systematic reviews. BMJ 2021;372:n71. doi: 10.1136/bmj.n71

For more information, visit: <http://www.prisma-statement.org/>
