# Supplementary material for: Meta-analysis of effects of yoga exercise intervention on sleep quality in breast cancer patients
Source: Front Oncol. 2023 Jun 30;13:1146433. doi: 10.3389/fonc.2023.1146433 (PMC10348890; doi:10.3389/fonc.2023.1146433)
Supplement: Supplementary file 2 [file DataSheet_1.doc]

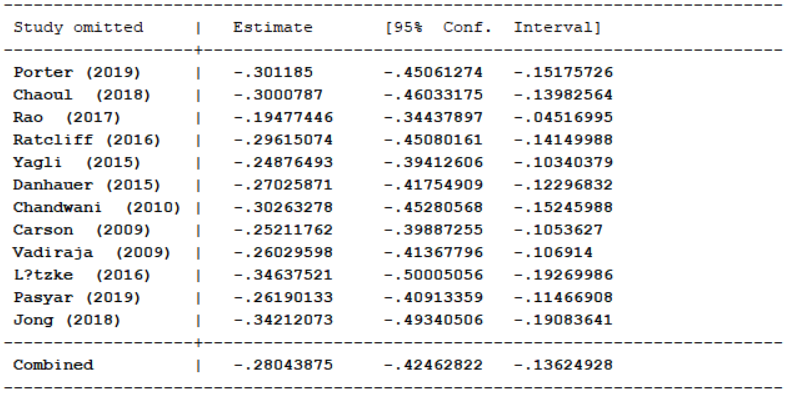


Fig 1 Funnel plot of Egger's method to assess publication bias(results).


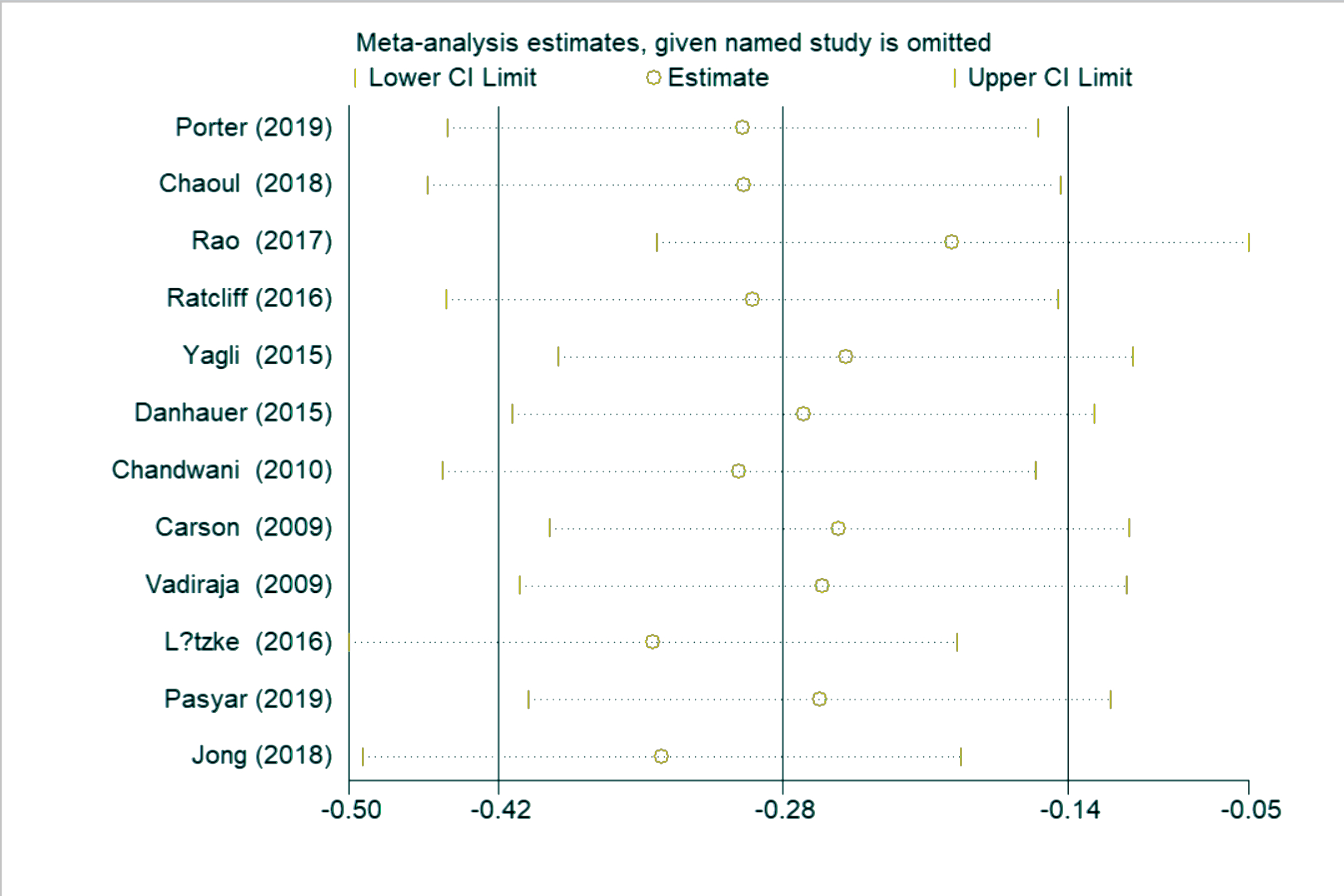


Fig 2 Funnel plot of Egger's method to assess publication bias(graph) .
